# The *Pm5e* Gene Has No Negative Effect on Wheat Agronomic Performance: Evidence From Newly Established Near-Isogenic Lines

**DOI:** 10.3389/fpls.2022.918559

**Published:** 2022-06-08

**Authors:** Dan Qiu, Jiang Huang, Guanghao Guo, Jinghuang Hu, Yahui Li, Hongjun Zhang, Hongwei Liu, Li Yang, Yang Zhou, Benzhou Yang, Yudan Zhang, Zhiyong Liu, Hongjie Li

**Affiliations:** ^1^The National Engineering Laboratory of Crop Molecular Breeding, Institute of Crop Sciences, Chinese Academy of Agricultural Sciences, Beijing, China; ^2^School of Intelligent Medicine and Biotechnology, Guilin Medical University, Guilin, China; ^3^State Key Laboratory of Plant Cell and Chromosome Engineering, Institute of Genetics and Developmental Biology, The Innovative Academy of Seed Design, Chinese Academy of Sciences, Beijing, China; ^4^College of Advanced Agricultural Science, University of Chinese Academy of Sciences, Beijing, China; ^5^College of Life and Environmental Sciences, Minzu University of China, Beijing, China; ^6^Institute of Wheat Sciences, Jining Academy of Agricultural Sciences, Jining, China

**Keywords:** *Triticum aestivum*, *Blumeria graminis* f. sp. *tritici*, *Pm5e*, resistance gene, agronomic traits

## Abstract

Wheat genotypes resistant to powdery mildew (*Blumeria graminis* f. sp. *tritici*, *Bgt*) provide a sustainable means for disease control. We developed a pair of near-isogenic lines H962R and H962S with contrasting reactions to powdery mildew from a residue heterozygous line. H962R was resistant to 127 out of the 136 *Bgt* isolates collected from the major wheat-producing regions of China and showed a similar virulence/avirulence pattern as Fuzhuang 30, Xiaobaidong, and Hongquanmang carrying resistance allele of *Pm5e*, but H962S was resistant to none of them. A dominant gene was responsible for the powdery mildew resistance of H962R as revealed by the genetic analysis using segregating populations derived from a cross between H962R and H962S. Molecular marker analysis detected a resistance locus, designated *PmH962*, on a genetic interval of the chromosome arm 7BL where *Pm5e* resides. This locus was co-segregated with the functional marker of *Pm5e*. The PCR-based sequence alignment of *Pm5e* demonstrated that H962R had an identical sequence as Fuzhuang 30 (haplotype HapGA), and H962S possessed the same sequence as the powdery mildew susceptible cultivar Kenong 199. The genomic compositions of lines H962R and H962S were highly comparable as evidenced by only a small percentage of SNP variations detected by the 16K Genotyping by Target Sequencing (GBTS) SNP array and the 90K Illumina iSelect Wheat SNP array. The two lines performed similarly in the yield-related and plant growth traits investigated, except for greater kernel weight in H962R than in H962S. This indicates that *Pm5e* has no deleterious effect and can be served as an excellent disease resistance gene in wheat breeding.

## Introduction

Wheat (*Triticum aestivum* L.) is exposed to various pathogen infections, among which *Blumeria graminis* f. sp. *tritici* (*Bgt*), the causal agent of wheat powdery mildew, is of global importance causing an estimated yield loss of below 10% in average and could be up to 30–40% when occurred severely (Singh et al., 2016; Savary et al., 2019; Bhavani et al., 2021). This fungal disease has been epidemic in China since the 1970s and currently occurs in an area of ca. 6–8 million hectares across both winter and spring wheat regions. In recent years, climate change enhances the spread of powdery mildew throughout the wheat fields across the country (Tang et al., 2017; Zhang et al., 2017).

The economic importance of powdery mildew promotes extensive research toward its sustainable management. The obligate biotrophic nature of the pathogen makes it survive only in alive up-ground tissues of plants such as leaves, stems, and spikes. So, it is feasible to restrict the multiplication and spread of the fungus with host resistance and, in turn, control epidemics of the disease. After several decades of efforts on gene mining research, more than 100 powdery mildew resistance genes or alleles with designation symbols of *Pm1* to *Pm68* and many others with temporal designations have been documented. Most of these genetic loci confer race-specific or all-stage resistance. Also, many quantitative trait loci (QTL), which are non-race specific and effective at the adult plant stage only, have been characterized (Kang et al., 2020).

Multiple alleles that occur in different wheat genotypes have been detected in several powdery mildew resistance loci. For example, locus *Pm5* has five alleles designated *Pm5a* through *Pm5e*, all of which are located on the long arm of chromosome 7B with a recessive or semi-dominant mode of inheritance. *Pm5a* was introgressed from Yaroslav Emmer (*Triticum dicoccum* Schübl) into an American wheat cultivar Hope (Lebsock and Briggle, 1974). Hsam et al. (2001) reported the characterization of *Pm5b*, *Pm5c*, and *Pm5d*. Also, they found the presence of *Pm5a* in several other wheat cultivars from different countries across Europe, North America, and Asia in addition to Hope. *Pm5b* in a series of German wheat cultivars Ibis, Aquila, Flanders, and Rothwell Perdix may originate from wheat line DHE516 from Hindu Kush Mountains (Bennett, 1984; Hsam et al., 2001). Kolandi, an accession of *Triticum sphaerococcum* var. *rotundatum* Perc., carries allele *Pm5c* (Hsam et al., 2001). *Pm5d* was transferred from a Chinese wheat landrace CI 10904 (PI 83395) into Sweden spring wheat cultivars Prins and Starke resulting in the production of lines IGV 1-455 and IGV 2-556 (Leijerstam, 1972; Hsam et al., 2001). *Pm5e* was originally detected in Fuzhuang 30 (Huang et al., 2003). Several alleles of *Pm5e* were identified in different Chinese wheat landraces. *Pm5e* encodes a nucleotide-binding domain leucine-rich-repeat (NLR) containing protein, and the functional markers were developed to facilitate its application in wheat breeding (Xie et al., 2020).

Despite powdery mildew resistance genes identified with clear positions on chromosomes and traced with molecular markers, not all of them are widely used in breeding. Breeders prefer to use the resistance genes with promising agronomic traits and no yield penalty. Any disease resistance genes must be accompanied by proper yield, quality, and agronomic traits, and even other disease and stress resistance, to ensure its successful application in breeding programs. Linkage and competitive drags are the major factors that determine the deployment of powdery mildew resistance genes in wheat breeding and production (Summers and Brown, 2013). Usually, pre-breeding steps are necessary to eliminate the deleterious genes that may link to the disease resistance genes of interest.

We developed a pair of near-isogenic lines with contrasting responses to powdery mildew from residue heterozygous lines. Line H962R was resistant to most *Bgt* isolates collected from China, in contrast to the susceptible line H962S. These lines provide a useful source of resistance in breeding, disclosure of disease resistance mechanism, and evaluation of the yield penalty of the disease resistance gene. This study was initiated to characterize their phenotypic reactions, genetic compositions, and genetic control of the disease resistance for these lines.

## Materials and Methods

### Plant Materials

H962 is an F_8_ line selected from a cross between wheat-*Thinopyrum ponticum* partial amphiploid Xiaoyan 693 (2*n* = 56) [pedigree: Xiaoyan 2/(Lin 7/*Th. ponticum*) F_1_] and common wheat line Ji87050 (pedigree: 72180/Jimai 30) (Li et al., 2003). This line was found to be heterozygous in the response to powdery mildew, and its residue heterozygous lines H962R (Resistant) and H962S (Susceptible) with distinct responses to powdery mildew were crossed to produce F_1_, F_2_ (461 plants), F_2:3_ (299 families), and F_2:9_ recombinant inbred line (RIL) (187 lines) populations for genetic analysis and molecular mapping of the powdery mildew resistance gene. Wheat genotypes with known powdery mildew resistance genes in the *Pm5* locus on the chromosome arm 7BL, including Hope (*Pm5a*), CI 14125 (*Pm5*c), Fuzhuang 30 (*Pm5e*), Hongquanmang (*PmH*), and Xiaobaidong (*mlxbd*), as well as another 17 wheat differential genotypes, were used to compare the reactions to different *Bgt* isolates from various wheat fields in China. Susceptible cultivars Zhongzuo 9504 and Chancellor were used to increase *Bgt* isolates and served as the susceptible control in all the powdery mildew tests. Selected Chinese Spring (CS) homoeologous group 7 nullisomic-tetrasomic lines (CSN7A-T7D, CSN7B-T7A, CSN7B-T7D, CSN7D-T7A, and CSN7D-T7), ditelosomic lines (CSDt7BS and CSDt7BL), and deletion lines (7BS-1, 7BL-6, 7BL-7, and 7BL-10) were used to determine the chromosomes on which the molecular markers linked to the powdery mildew resistance gene in H962R reside.

### Agronomic Trait Evaluation

A field trial was conducted in a farm of the Institute of Crop Sciences, Chinese Academy of Agricultural Sciences (CAAS), Beijing (116°249′ E, 40°179′ N) during the cropping seasons of 2010–2011. Lines H962R and H962S were arranged in a randomized complete block design with three replicates. Each plot consisted of three rows of 2 m in length and 0.3 m between rows. Dates from sowing to heading, anthesis, and maturity were recorded on a plot basis. Ten plants from each plot were randomly harvested to investigate plant height, spike, and kernel traits, such as spike length, number of spikelets per spike, number of kernels per spike, thousand-kernel weight, glume, and kernel color (Supplementary Table 1).

### Powdery Mildew Assessments

The *Bgt* isolates were originally collected from wheat fields of Shandong, Hebei, Henan, Beijing, and other provinces (Supplementary Table 2). They were subjected to at least three rounds of single-pustule culture prior to the preparation of inocula for disease assessments. A total of 136 isolates were applied in the seedling tests. Two-leaf stage seedlings grown in plastic pots (5 × 5 × 5 cm in dimension) were dusted with freshly increased conidiospores, incubated in dew plastic bags for 24 h in the dark, and allowed for disease symptom development for 2 weeks in a greenhouse set at 20 ± 2°C, 80% relative humidity, and a photoperiod of 14 h light/10 h dark. The phenotypic categorization of wheat plants was carried out based on the infection types (ITs) of the primary leaves rated on a 0–4 scale. Wheat plants were regarded to have a resistant phenotype when their ITs were 0 (immune), 0; (hypersensitive reaction), 1 (highly resistant), and 2 (moderately resistant), or to have a susceptible phenotype when their ITs were 3 (moderately susceptible) and 4 (highly susceptible) (Liu et al., 1999). At least two parallel tests for each wheat genotype were tested.

### Analysis of Genomic Compositions for Lines H962R and H962S

Total genomic DNA of H962R and H962S was extracted with a Hi-DNA secure Plant Kit (DP350, Tiangen Biotech Co., Beijing, China). After examination of quality and concentration using an agarose gel electrophoresis and NanoDrop One (Thermo Fisher Scientific, Waltham, MA, United States), DNA samples of the two lines were genotyped with the 16K Genotyping by the Target Sequencing (GBTS) SNP array and the 90K Illumina iSelect Wheat SNP array (MolBreeding Biotechnology Co., Ltd., Shijiazhuang, China). Raw reads generated by the 16K GBTS SNP array were filtered using fastp software (version 0.20.0, parameter: -n 10 -q 20 -u 40). Clean reads were aligned to the Chinese Spring genome sequence using Burrows-Wheeler Aligner (BWA) software (Li and Durbin, 2009). The UnifiedGenotyper and VariantFiltration modules in GATK software were used for mutation detection and data filtering. Software ANNOVAR was used for functional annotation of the gene mutations detected. For the 90K SNP array (Wang et al., 2014), SNP calling was carried out in the Illumina iScan reader with the Genome Studio software (version 2011.1; Illumina Inc., San Diego, CA, United States). The genotypic clusters for each SNP were determined based on the data for the two lines using the manual option of Genome Studio software. The diagrams of chromosomal compositions for the two lines were drawn by the self-written program in the R package (MolBreeding Biotechnology Co., Ltd., Shijiazhuang, China).

As Xiaoyan 693 and 72180 have *Th. ponticum* in their pedigree (Zhang et al., 1989; Li et al., 2003), the presence of *Th. ponticum* chromatin in the H962 lines, if any, was detected using genomic *in situ* hybridization (GISH) and the *Thinopyrum* genome-specific marker analyses as described previously (Li et al., 2004). The mitotic chromosomes were prepared from root tip cells for GISH analysis. The total genomic DNA from *Th. ponticum* was labeled with biotin-14-dATP as a probe, and the total genomic DNA from Chinese Spring wheat was sheared as a blocker. The hybridization signals were visualized under a fluorescent microscope (Carl Zeiss, Oberkochem, Germany) after staining with fluorescein isothiocyanate (FITC)-avidin DN and biotinylated goat anti-avidin D (Vector Laboratories, Inc., Burlingame, CA, United States). Propidium iodide was used to counterstain the wheat chromosomes. A pair of primers, 2P1 and 2P2, which was derived from a repetitive sequence and can specifically detect chromatin of genus *Thonopyrum* (Wang and Wei, 1995), was used to detect *Th. ponticum* genome-specific DNA. Since the parental cultivar Jimai 30 is a T1BL⋅1RS translocation cultivar (Wei et al., 2013), markers ω-sec-P1 and ω-sec-P2 specific for the ω-seclin gene on the chromosome arm 1RS of rye (*Secale cereal* L.) were used to detect this translocation (Froidmont, 1998). The amplification products in a Biometra T3000 Thermocycler (Applied Biosystems, New York, NY, United States) were visualized on a 1.5% agarose gel.

### Molecular Marker Analysis

Polymorphisms of the WMC, MVG, GWM, CFD, and BARC series of SSR markers and the EST-based markers on chromosome arm 7BL were determined using H962R and H962S. To develop more markers, RNA sequencing was performed on an Illumina Hi2500 platform (BGI, Shenzhen, China) with leaves of H962R and H962S at 0, 4, 8, 16, 24, 48, and 72 h post-inoculation (hpi) with *Bgt* isolate E09. Raw reads were purified by trimming adapters and removing low-quality sequences. Adaptor-trimmed reads comprising 40,349 unigene sequences with a total of 31,671,110 bases were mapped to *T. aestivum* genome sequence (International Wheat Genome Sequencing Consortium [IWGSC], 2018). The number of clean reads in each library was normalized to the Transcripts Per kilobase of exon model per Million (TPM) mapped reads to obtain the normalized gene expression level. The differentially expressed genes (DEGs) between H962R and H962S were determined with the parameters of false discovery rate (FDR) ≤ 0.001 and threshold absolute log_2_-fold change ≥ 1 for the sequence counts across the libraries using DESeq2.^1^ Sequences of the DEGs were used to design primer pairs using online website Primer3Plus.^2^

Ten RILs from cross H962R × H962S with contrasting responses to each powdery mildew were separately pooled to construct the resistant and susceptible DNA bulks for bulked segregant analysis (BSA). Each reaction mixture (10 μl) consisted of 10 mM Tris-HCl (pH 8.3), 50 mM KCl, 1.5 mM MgCl_2_, 250 μM each of the dNTP, forward and reverse primer 0.2 μM each, 0.5 U *Taq* DNA polymerase, and 50 ng DNA template. Amplification of DNA was programmed at 94°C for 5 min, 38 cycles of 94°C for 35 s, 50–62°C for 35 s, and 72°C for 1 min, and a final extension at 72°C. Products amplified were separated on 8% non-denatured polyacrylamide gels with a 29:1 of acrylamide and bisacrylamide and visualized by silver staining. Polymorphic markers were used to genotype the RIL population to determine the linkage with the target gene. The KASP marker Pm5e-KASP, which was designed based on the sequence of *Pm5e* (Xie et al., 2020), was also used to genotype the RIL population to examine the linkage between *Pm5e* and the powdery mildew resistance in H962R.

### Alignment of *Pm5e* Sequences

Total genomic DNA was extracted using a Hi-DNA secure Plant Kit (DP350, Tiangen Biotech Co., China) for amplifying the *Pm5e* sequences with primers as listed in Supplementary Table 3. The resulting sequences from H962R and H962S were aligned to the *Pm5* allelic sequences from Fuzhuang 30 (*Pm5e*, HapGA), Baimangduomai (*PmBMDM*, HapGG), Chinese Spring (*Pm5-CS*, HapCG), Non-gda 015 (*Pm5-ND*, HapCG), Hope (*Pm5a*), and Mission (*Pm5b*), as well as the wheat 10 + genomes (Walkowiak et al., 2020), Kenong 199 (*Pm5-KN199*), and Fielder (Sato et al., 2021). Multiple sequence alignment was performed using Snapgene (version 4.1.9),^3^ and the TriticeaeGeneTrebe (TGT) website was used for homologous gene search, structure alignment, and clustering.^4^

### Statistical Analysis and Genetic Map Construction

All statistical analyses were performed using the SAS package (Version 9.2, SAS Institute, Cary, NC, United States). ANOVA for the agronomic traits was carried out to test the significance of the differences between H962R and H962S using the least significant difference (LSD) at *P* < 0.05. The IT values were recorded as a data matrix composed of 1 (resistant) and 2 (susceptible). A dendrogram was drawn using the online website HIPLOT,^5^ to compare the relationship between the target gene and the known powdery mildew resistance genes. The chi-squared test (χ^2^) was performed to determine the goodness of fit for the observed data from the expected segregation ratios of the segregating populations. The linkage relationship between the polymorphic markers identified by BSA and the target resistance gene was determined with Mapmaker Version 3.0 software (Lincoln et al., 1993). The genetic distance between markers and the target gene was calculated using the Kosambi function (threshold LOD ≥ 3.0), and the maximum linkage distance was set at 50.0 (Kosambi, 1943). The genetic linkage map was constructed by Mapdraw (Version 2.1) (Liu and Meng, 2003).

## Results

### Development of Isogenic Lines H962R and H962S

Wheat-*Th. ponticum* partial amphiploid Xiaoyan 603 (2*n* = 56) was crossed with wheat line Ji87050 in 1989 and self-pollinated repeatedly to obtain line H962 from the F_8_ progenies. This line was segregated for powdery mildew reactions when inoculated with *Bgt* isolate E03, and the resistant and susceptible plants were subjected to nine generations of self-pollination. Two near-isogenic lines, designated H962R (Resistant) and H962S (Susceptible), were finally obtained for characterization of their genomic composition and genetic control of disease resistance.

### Characterization of Genomic Compositions for H962R and H962S

**FIGURE 1:**
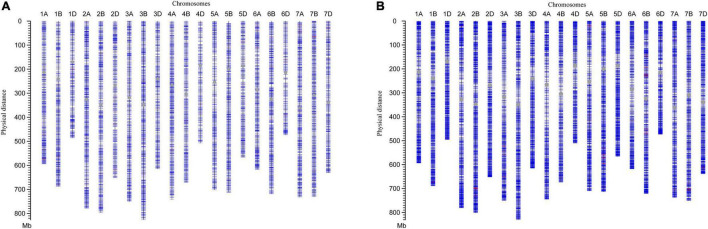
Diagram of chromosomal compositions for H962R and H962S by 16K GBTS SNP array **(A)** and 90K Illumina iSelect Wheat SNP array **(B)**. The blue and red lines represent the same and different SNPs between H962R and H962S, respectively. The centromeres are indicated by the gray dots.

Two versions of the SNP array were applied to characterize the genomic compositions of lines H962R and H962S. The majority of SNP loci (14,315 out of 14,387, 99.5%) were common between the two lines generated by the 16K GBTS SNP array. Seventy-two (0.5%) polymorphic SNP loci were scattered on different wheat chromosomes with similar numbers in the A (22), B (26), and D (24) genomes (Figure 1A). Similarly, the 90K Illumina iSelect Wheat SNP array identified 136 (0.2%) polymorphic loci among 79,955 SNP loci detected (Figure 1B). These results demonstrate the high level of genomic similarity between H962R and H962S.

The GISH with the *Th. ponticum* genomic DNA as a probe and Chinese Spring genomic DNA as a blocker detects a complete set of 42 wheat genome chromosomes without any *Th. ponticum* chromosomes or chromosome segments in H962R and H962S (data not shown). Meanwhile, *Thinopyrum* genome-specific primers 2P1 and 2P2 did not amplify any *Thinopyrum* chromatin in the H962 siblings (Supplementary Figure 1). However, the wheat-rye translocated chromosome T1BL⋅1RS was detected in both H962R and H962S *via* molecular markers specific for the rye chromosome arm 1RS (Supplementary Figure 2).

### Seedling Reaction Patterns of H962R and H962S to *Bgt* Isolates

**FIGURE 2:**
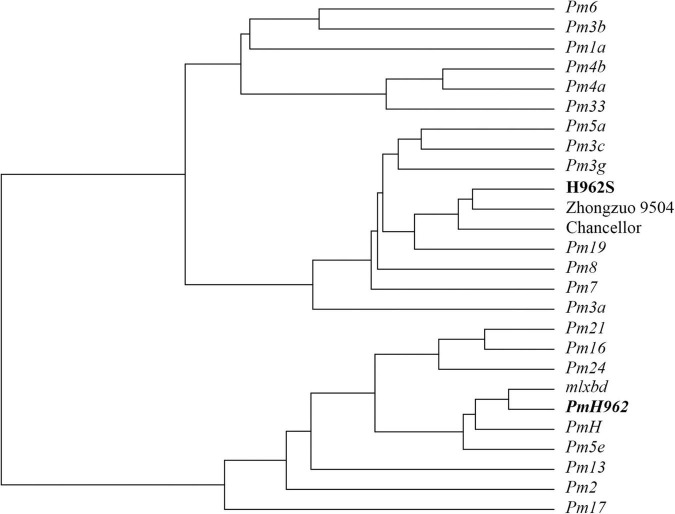
A dendrogram based on reactions of *PmH962* and differential wheat cultivars/lines possessing known powdery mildew resistance genes against 103 *Blumeria graminis* f. sp. *tritici* isolates.

An array of 103 *Bgt* isolates collected from different provinces during 2011–2016 were used to compare the seedling reaction patterns between the two H962 lines. These isolates showed different virulence patterns against 22 wheat accessions carrying different known powdery mildew resistance genes (Supplementary Table 2). H962R was resistant to 94 *Bgt* isolates, while H962S was as susceptible to all of them as that of the susceptible controls Zhongzuo 9504 and Chancellor. The virulence/avirulence patterns of H962R to these *Bgt* isolates were most similar as those of Fuzhuang 30 (*Pm5e*), Xiaobaidong (*mlxbd*), and Hongquanmang (*PmH*), which carry the *Pm5e* locus (Figure 2 and Supplementary Table 2). The reactions of H962R differed from these *Pm5e*-carrying genotypes in only a few isolates. Another 33 isolates recently collected during 2018–2020 were also tested, and all of them were avirulent on H962R but virulent on H962S (Supplementary Table 4).

### Inheritance of the Powdery Mildew Resistance in H962R

**FIGURE 3:**
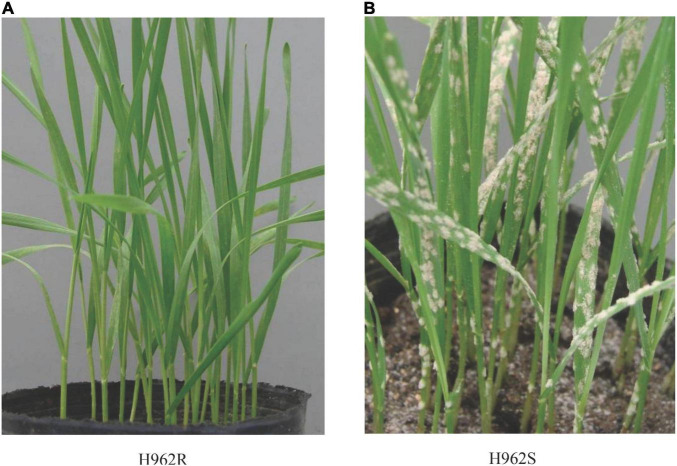
Seedling phenotypes of H962R **(A)** and H962S **(B)** to *Blumeria graminis* f. sp. *tritici* isolate A49.

When challenged with *Bgt* isolate E09 at the seedling stage, H962R (IT 0) was distinct from H962S (IT 4) in the response to powdery mildew (Figure 3). So, this isolate was used to determine the inheritance of powdery mildew resistance using the populations developed from the cross between H962R and H962S. The phenotypic segregation patterns for the resistant and susceptible plants (or lines) in the F_1_ (1:0), F_2_ (3:1), F_2:3_ (1:2:1), and F_2:9_ (1:1) populations clearly demonstrated that a single dominant gene, designated *PmH962*, governs the resistance to *Bgt* isolate E09 in H962R (Table 1).

**TABLE 1:** Inheritance analysis of lines H962R and H962S and their F_1_, F_2_, F_2:3_, and F_2:9_ progenies for resistance to *Blumeria graminis* f. sp. *tritici* isolate E09.

Parents or cross	Generation^a^	Total number of plants/families	Observed ratio		Expected ratio	χ^2^	*P*-value
			
			R	S			
H962R	P_R_	28	28				
H962S	P_S_	30		30			
H962R × H962S	F_1_	20	20				
H962R × H962S	F_2_	461	346	116	3:1	0.003	0.957
H962R × H962S	F_2:3_	117	A:H:B = 32:57:28^b^		1:2:1	0.350	0.839
H962R × H962S	F_2:9_	187	A:B = 97:90		1:1	0.262	0.609

*^a^P_R_, resistant parent; P_S_, susceptible parent.*

*^b^A, homozygous resistant; H, segregating (heterozygous resistant); B, homozygous susceptible.*

### Identification of SSR and EST-SSR Markers Linked to *PmH962*

Due to high similarity in the genomic compositions between the two lines, only about 6% of 1,380 SSR and EST markers screened were polymorphic between H962R and H962S. Two markers *Xgwm783* and *XTC17* (Supplementary Table 3) anchored on chromosome 7BL were further polymorphic between the resistant and susceptible DNA bulks. After genotyping the F_2:3_ population, they were linked to *PmH962* with genetic distances of 8.3 and 6.4 cM, respectively. The examination of additional 180 EST-derived markers on chromosome 7BL identified another *PmH962*-linked marker *Xics-X27* (Supplementary Table 3).

### Development of Molecular Markers From Differentially Expressed Genes Generated by RNA-Seq Analysis

**FIGURE 4:**
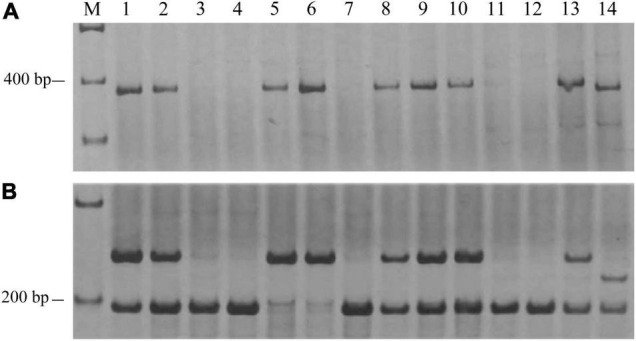
The banding patterns of *PmH962*-linked markers *NL2* **(A)** and *XTC17* **(B)** in the Chinese Spring (CS) homoeologous group 7 nulli-tetrasomics, ditelosomics, and deletion lines. 1, CS; 2, CSN7A-T7D; 3, CSN7B-T7A; 4, CSN7B-T7D; 5, CSN7D-T7A; 6, CSN7D-T7B; 7, CSDt7BS; 8, CSDt7BL; 9, 7BS-1; 10, 7BL-6; 11, 7BL-7; 12, 7BL-10; 13, H962R; 14, H962S.

Due to the low polymorphism for the available SSR markers between lines H962R and H962S, RNA-Seq was applied to isolate E09-inoculated leaves of the two lines to identify the DEGs for developing more polymorphic molecular markers. The statistical results of the RNA-Seq are shown in Supplementary Table 5. The 155 top DEGs were selected and 12 of them were anchored on chromosome 7BL. Sequences of these DEGs were used to design 188 gene-specific primer pairs. Markers *Xics-NL2* and *Xics-X5*, specific for genes *TraesCS7B01G441600.1* and *TraesCS7B01G441700.1* (Supplementary Table 3), respectively, were polymorphic and linked to *PmH962* after examining the contrasting DNA bulks constructed with the RIL population. Physical mapping of markers *Xics-NL2* and *XTC17* with the Chinese Spring nulli-tetrasomic lines, ditelosomic lines, and deletion lines for the homoeologous group 7 chromosomes indicated that they were located on bin 7BL10-0.78-1.00 at the end of chromosome 7BL (Figure 4). Although the wheat-rye translocated chromosome T1BL⋅1RS carrying *Pm8* was present in both H962R and H962S, this translocation was not associated with the resistance of H962R to powdery mildew as evidenced by 1RS-specific marker genotyping the RIL population of cross H962R × H962S (data not shown).

### Sequence Characteristics of *Pm5e* in H962R and H962S

**FIGURE 5:**
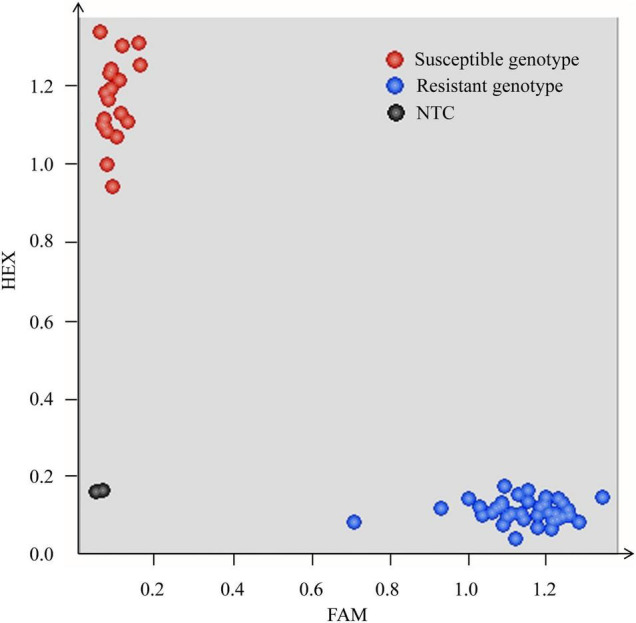
Scatter plots of the RIL population from cross H962R × H962S for the Kompetitive allele-specific PCR (KASP) assay using the *Pm5e* functional marker Pm5e-KASP. The blue and red dots represent the lines with the disease-resistant and susceptible genotypes, respectively. The non-template control is indicated by the black dots.

Since marker *Xics-X5* was derived from gene *TraesCS7B01G441700*, which was annotated as the *NLR* gene, *Pm5e*, the functional marker of this cloned gene, Pm5e-KASP, was used to genotype the RIL population of cross H962R × H962S. The result confirmed the co-segregation of Pm5e-KASP with the powdery mildew resistance in this population (Figure 5).

**FIGURE 6:**
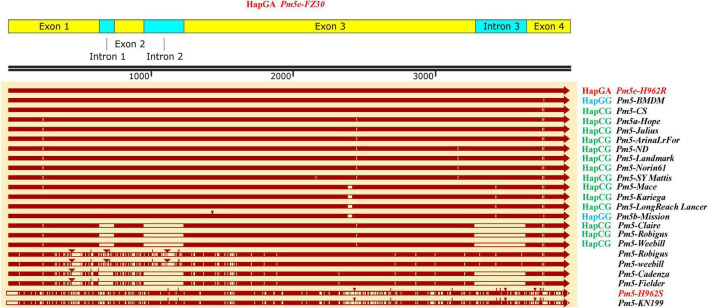
Sequence alignment of *Pm5e* from different genotypes.

The haplotypes of H962R and H962S at the *Pm5* locus were compared based on their sequences. The amplified sequence of *Pm5e* from H962R (3,948 bp) was identical with that from Fuzhuang 30, belonging to the resistant haplotype HapGA (Figure 6). It differed from the susceptible haplotypes HapCG from Chinese Spring, Hope (*Pm5a*), Julius, ArinaLrFor, Landmark, Norin61, SY Mattis, Mattis, Mace, Kariega, LongReach Lance, Claire, Robigus, and Weebill, as well as haplotype HapGG, represented by Mission (*Pm5b*). The *Pm5e* allelic sequence from H962S (3,980 bp) shared the same sequence as Kenong 199 and had 91% sequence identity compared with H962R with many insertions, deletions, and SNP variations.

### Impacts of *Pm5e* on Agronomic Performance

**FIGURE 7:**
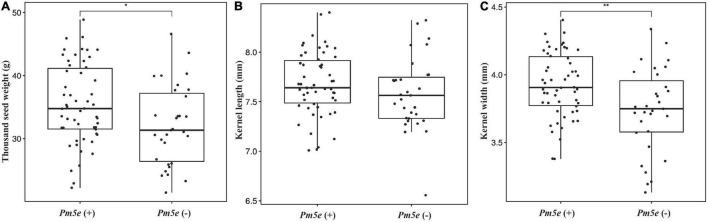
Box plots of thousand-kernel weight **(A)**, kernel length **(B)**, and kernel width **(C)** for the lines with or without *Pm5e* from the cross H962R × H962S.

Line H962R (51.30 ± 0.39 g) produced greater thousand-kernel weight than H962S (45.84 ± 0.62 g), with a significant difference of 5.46 ± 0.94 (*P* < 0.05). Examination of the RIL population of H962R × H962S revealed that the lines carrying *Pm5e* had a mean thousand-kernel weight of 35.46 ± 6.34 g with a range of 22.23–48.84 g, which was significantly greater than that of the lines without *Pm5e* (32.12 ± 6.34 g, with a range of 19.20–47.04 g) (Figure 7). A significant difference in kernel width but not in kernel length was observed between the two groups of RILs. Lines H962R and H962S did not differ in several other spike and kernel traits, e.g., number of spikelets and kernels per spike, as well as glume and kernel colors (Supplementary Table 1). The two near-isogenic lines were highly comparable in the plant traits, such as dates from sowing to heading, flowering, maturity, and plant height.

## Discussion

We developed a pair of near-isogenic lines, H962R and H962S, from the residue heterozygous line of H962, a progeny originating from a cross between wheat-*Th. ponticum* partial amphiploid Xiaoyan 693 and common wheat line Ji87050. H962R and H962S possess nearly identical genomic constitutions, as evidenced by the small proportions of SNP variations detected by the 16K GBTS and 90K iSelect SNP arrays. However, the two lines were distinct in their reactions to powdery mildew. Line H962R was resistant to most *Bgt* isolates collected in a wide range of wheat fields in China, while H962S was resistant to none of them. Genetic and molecular mapping identified a dominant gene *PmH962* on the chromosome arm 7BL that is responsible for its disease resistance. This gene was mapped to the genomic region where *Pm5e* resides and was co-segregated with the *Pm5e* functional marker Pm5e-KASP. H962R shared the same sequence of *Pm5e* and performed similar virulence/avirulence patterns to an array of *Bgt* isolates as Fuzhuang 30, the *Pm5e* carrier. H962S had an identical sequence as the susceptible cultivar Kenong 199 at the *Pm5* locus. Collectively, the powdery mildew resistance of H962R is conferred by gene *Pm5e*.

*Pm5e* was initially detected in Fuzhuang 30, a cultivar selected from Jinghui 30 that was developed from a cross between two Chinese wheat landraces Liquanheshangtou and Huaxianqisifeng in Shaanxi Province in the 1940s (Huang et al., 2003). Subsequent studies identified several genes allelic to *Pm5e* in wheat landraces, e.g., Hongquanmang (*PmH*) (Zhou et al., 2005), Hongyoumai (*pmHYM*) (Fu et al., 2017), Baiyouyantiao (*PmBYYT*) (Xu et al., 2018a), Shangeda (*PmSGD*) (Xu et al., 2018b), Dahongtou (*pmDHT*) (Qie et al., 2019), Youbailan (*pmYBL*) (Xu et al., 2020), Xiaobaidong (*mlxbd*) (Xue et al., 2009), and Duanganmang (*PmDGM*) (Wu et al., 2021). Most of these landraces were ever grown in the Guanzhong Plain, Shaanxi Province, or nearby regions (Xie et al., 2020). The parents of H962, 72180 and Xiaoyan 693, were also developed in Shaanxi Province (Zhang et al., 1989). Although both of them have *Th. ponticum* in their pedigrees, GISH and genome-specific PCR analyzes did not detect any evidence that the H962 lines possessed any *Thinopyrum* chromatin. Therefore, it is reasonable to speculate that the origin of the *Pm5* allele in H962R is derived from common wheat grown in the region where the other *Pm5e*-carrying landraces originated.

Most alleles of the *Pm5* locus perform a recessive mode of inheritance, as was observed in the majority of wheat landraces known to carry the *Pm5e* alleles. Exceptions are cultivar Tangmai 4 and landrace Duanganmang with the dominant alleles. Sequence analysis confirms that both of them carry *Pm5e* (Xie et al., 2017, 2020; Wu et al., 2021). Genetic analysis of the powdery mildew resistance also revealed a dominant inheritance in line H962R, which also possesses *Pm5e*. The genomic region that flanks *Pm5e* contains several NLR genes (Xie et al., 2020). This indicates that there might exist uncharacterized genetic factors that coordinate the expression of *Pm5e* in different genetic backgrounds. Although the T1BL⋅1RS chromosome translocation was present in both H962R and H962S, *Pm8* on this chromosome appears to be unlikely to affect the phenotypes of powdery mildew resistance. More studies are needed to elucidate the different expression patterns of *Pm5e* in the H962 sib-lines and other *Pm5e* carriers.

A broad spectrum of disease resistance has been observed in most *Pm5e* carriers including H962R. These *Pm5e*-carrying wheat landraces had once been used in agriculture and did not have deleterious traits. For example, the original *Pm5e* carrier, Fuzhuang 30, and its parental cultivar Jinghui 30 were widely grown in Shaanxi Province (Xie et al., 2020). To date, these landraces are not grown due to their poorer productivity. Because the functional marker of *Pm5e* is available, it is easy to incorporate these *Pm5e* alleles into modern wheat breeding programs with the aid of molecular marker-assisted selection.

Success of powdery mildew resistance in breeding for resistant wheat cultivars depends on not only its disease resistance but also the backgrounds in which it resides. For example, *Pm16* failed to be deployed in commercial wheat cultivars due to a significant yield penalty (Summers and Brown, 2013). Gene *Pm5e* in line H962R did not link to deleterious genes for poor agronomic traits, as revealed by the similar performances in most traits associated with growth and yield-related parameters observed. Moreover, the RILs of the H962R × H962S that carry *Pm5e* had greater kernel weight than those without *Pm5e* in the field without powdery mildew pressure, although we cannot determine whether *Pm5e* is linked to gene or QTL for kernel weight yet. This provides further evidence that *Pm5e* does not exert negative effects on wheat genotypes and has great potential in wheat breeding.

## Data Availability Statement

The data presented in the study are deposited in the National Genomics Data Center repository (https://bigd.big.ac.cn/gsa/browse/CRA006702), accession number: CRA006702.

## Author Contributions

HJL and ZL conceived and designed the research. DQ, JAH, GG, JHH, YL, HZ, HWL, LY, YZ, BY, and YDZ conducted the experiments and analyzed the data. HJL, DQ, and ZL wrote the manuscript. All authors contributed to the article and approved the submitted version.

## Conflict of Interest

The authors declare that the research was conducted in the absence of any commercial or financial relationships that could be construed as a potential conflict of interest.

## Publisher’s Note

All claims expressed in this article are solely those of the authors and do not necessarily represent those of their affiliated organizations, or those of the publisher, the editors and the reviewers. Any product that may be evaluated in this article, or claim that may be made by its manufacturer, is not guaranteed or endorsed by the publisher.

## Footnotes

1
https://bioconductor.org/packages/DESeq2/

2
https://www.primer3plus.com/

3
https://www.snapgene.com/

4
http://wheat.cau.edu.cn/TGT/

5
https://hiplot.com.cn

## Supplementary Material

Supplementary Figure 1 Detection of *Th. ponticum* gene using primer 2P1 and 2P2. M: 100 bp DNA ladder; 1: Xiaoyan 693; 2: H962R; 3: H962S.

Supplementary Figure 2 Detection of *Th. ponticum* gene using primer ω-sec-P1 and ω-sec-P2. M: 100 bp DNA ladder; 1: H962R; 2: H962S; 3 and 4: *Secale cereale*; 5: 1R-2; 6: 1RL.
