# Supplementary figures and images for: The Pm5e Gene Has No Negative Effect on Wheat Agronomic Performance: Evidence From Newly Established Near-Isogenic Lines
Source: Front Plant Sci. 2022 Jun 8;13:918559. doi: 10.3389/fpls.2022.918559 (PMC9216190; doi:10.3389/fpls.2022.918559)

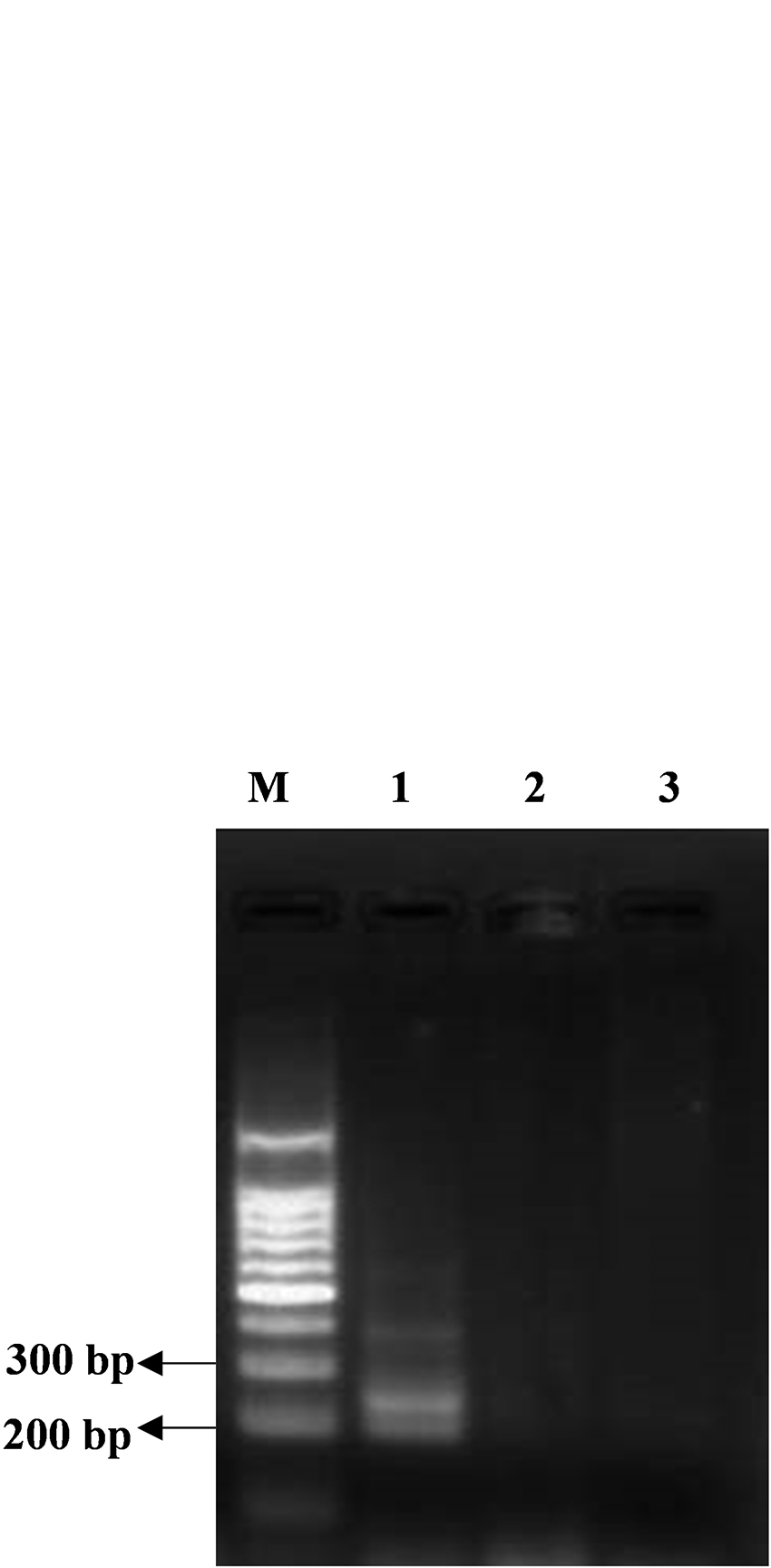

Supplement: Supplementary Figure 1 — Detection of Th. ponticum gene using primer 2P1 and 2P2. M: 100 bp DNA ladder; 1: Xiaoyan 693; 2: H962R; 3: H962S. [file Image_1.TIFF]

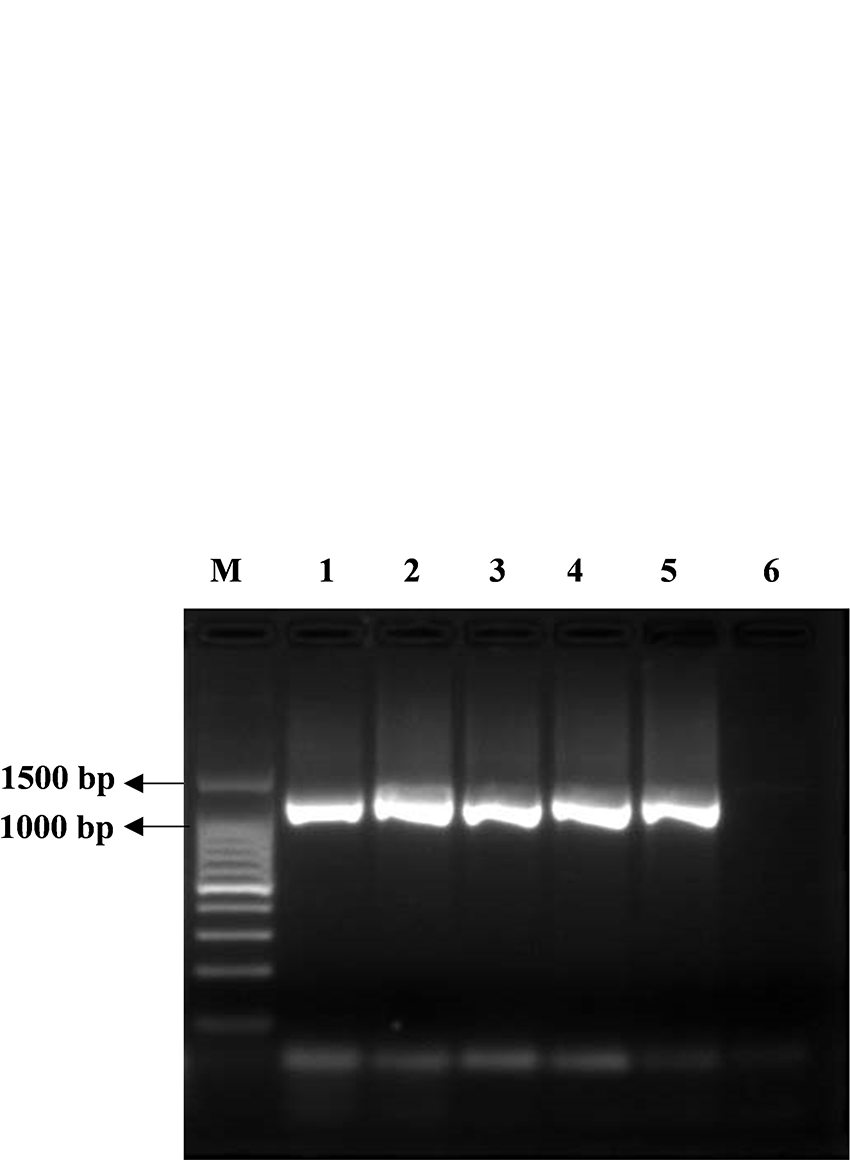

Supplement: Supplementary Figure 2 — Detection of Th. ponticum gene using primer ω-sec-P1 and ω-sec-P2. M: 100 bp DNA ladder; 1: H962R; 2: H962S; 3 and 4: Secale cereale; 5: 1R-2; 6: 1RL. [file Image_2.TIFF]
